# Well-being and associated factors among adults in the occupied Palestinian territory (oPt)

**DOI:** 10.1186/s12955-016-0519-2

**Published:** 2016-08-30

**Authors:** Nouh Harsha, Luay Ziq, Rula Ghandour, Rita Giacaman

**Affiliations:** 1 Ramallah, West Bank Palestine; 2 UNRWA, Birzeit, Ramallah, West Bank Palestine; 3 Institute of Community and Public Health, Birzeit University, Ramallah, West Bank Palestine

**Keywords:** Well-being, Standard of living, Community participation, Religious activities, Palestine

## Abstract

**Background:**

The World Health Organization (WHO) incorporated well-being into its definition of health in 1948. The significance given to this concept is due to its role in the assessment of people’s quality of life and health.

**Methods:**

Using the WHO Well-being Index, we estimated well-being among adults and identified selected associated factors in the occupied Palestinian territory (oPt) using data obtained from the National Time Use Survey conducted by the Palestinian Central Bureau of Statistics (PCBS) 2012–2013 on a representative sample of persons living in the West Bank and Gaza Strip. Univariate and bivariate analyses were conducted among participants 18 years old and above. Multivariate analysis (Regression) was performed with factors found significant in cross-tabulations, using SPSS® version 20.

**Results:**

Overall, 33.8 % (2395) of respondents reported low levels of well-being (ill-being). Neither age, nor sex, nor region were found significant in regression analysis. People who were married, working 15 h or more, with a higher standard of living, who reported participating in community, cultural, and social events, or in religious activities reported high levels of well-being. Those who reported regularly following the mass media, or living in Palestinian refugee camps reported low levels of wellbeing.

**Conclusions:**

Overall, about one-third of adult Palestinians reported low levels of well-being (ill-being), a finding which in itself requires attention. Marriage, employment, high living standards, community participation, and religious activities were found to be protective against ill-being. Further investigations are required to determine additional causes of ill-being in the oPt, taking into consideration the possible effects of chronic exposure to political violence on subjective well-being.

## Background

Well-being is a broad, complex phenomenon, that can be defined as “peoples’ positive evaluations of their lives” [1]. In 1948, well-being was incorporated into the broad definition of health that encompassed “complete physical, mental, and social well-being” [2]. Recently, well-being has received increasing attention from epidemiologists, economists, psychologists, behavioral and social scientists, philosophers, policy makers, and has even become a part of public policy discourse [3–7].

Subjective well-being consists of two main components: cognitive and affective [8]. The cognitive element is related to evaluation and judgment of people of their own lives including aspects such as work satisfaction, and life satisfaction; that is, specifically chosen criteria assessing the quality of life of persons [3]. The affective dimension deals with moods, feelings, and emotions [9]. The latter dimension entails two main elements: positive emotions like happiness, affection, joy, giving rise to pleasant feelings and a positive mood; and negative emotions like sadness, anger, stress, which are responsible for negative mood, and unpleasant feelings [6, 10].

Subjective well-being is affected by both internal as well as external factors [4]. Indeed, researches indicate that genetic factors, the early environment where people grow up, personality (e.g., optimism, intelligence), demographic factors (age, sex, education, marital status, and employment), social factors (social capital, social networks, social security, type of the government, presence of law, income inequality among people, religious activities, freedom, and human rights), socioeconomic conditions (people ranking within the society), and health status have an important impact on well-being [6, 8].

In the Palestinian context, the protracted warlike conditions with periods of acute intensification have been endured by Palestinians for almost a century, with chronic exposure to political violence adding to the burdens of people’s daily lives. To be sure, the lack of political stability deters sustainable economic development, a necessary aspect affecting the wellbeing of the population [11]. In addition, restrictions on the freedom of movement, human rights violations, inability to fulfill material needs, fragmentation of health services, and economic instability are likely to prevail at the time of wars and conflict, and can negatively affect well-being and life quality [12, 13].

This study aimed to assess the levels of well-being/ ill-being in Palestine using data from the National Time Use Survey collected by the Palestinian Central Bureau of Statistics in 2012–2013, and to identify factors associated with well-being/ill-being among adult Palestinians. We hypothesize that prevalence of ill-being in the occupied Palestine territory is relatively high.

## Method

The sample contained in the National Time Use Survey 2012–2013 covered all 16 governorates of the West Bank and Gaza Strip and urban, rural and refugee camp locales, and is representative of all of the population 10 years or more. A total of 5903 households were included in the survey. The response rate was 79.6 %. The questionnaire was filled by 8560 respondents [14].

### Measures

For this paper, we built two scales: Well-being and standard of living. The Well-being scale is composed of five questions (Appendix) contained in the World Health Organization’s Well-Being index [15]. This scale has been validated, translated to many languages, and used in studies in different countries [16]. Reliability and consistency were checked, Cronbach’s Alpha was 0.81, which indicates very good internal consistency/reliability. A six-point scale (1 = Always, 2 = More than often, 3 = Slightly more than half the time, 4 = Slightly less than half the time, 5 = A little bit of time, 6 = No, never) was used. Answers were recoded from 0 (no, never) to 5 (always). The scale was multiplied by 4 to obtain a 100-point scale. Then it was recoded into two categories: low levels of well-being (ill-being), and moderate to high levels of well-being, using 0.5 as the cutoff point, since it is commonly used in the literature, in practice, and recommended internationally [17].

The Standard of Living scale included ten items (Appendix) or household assets. The items were (private car, satellite TV, solar heating of water, vacuum cleaner, home library, PC, Palestinian internet services line, microwave, DVD player, and air condition). Reliability and consistency were checked, and Cronbach’s Alpha was 0.74, which indicates good internal consistency/reliability, with 0 (no), and 1 (yes) reported included. The scale was then recoded into low standard of living (having two item or less), medium standard of living (having 3 to 6 items), and high standard of living (having seven items or more) in a distribution similar to those reported by PCBS [18].

### Statistical analysis

Data were checked for accuracy, and cleanliness. Data analysis was performed using questionnaires completed by adults 18 years and above to avoid likely errors that may occur in some of the independent variables such as marriage, and labor status. Well-being was used as the dependent variable. Independent variables included demographic factors (age, sex, marital status), labor/employment status, standard of living, community participation, religious activities, use of the mass media, region, and type of locality. Descriptive statistics was completed for all the independent variables. Bivariate analysis was then conducted to assess the association of the independent variables with well-being, using the Chi Square test. Binary logistic regression was completed with independent variables found significant in the bivariate analysis. Regression results were confirmed with One-way ANOVA and linear regression, with no major differences found between linear regression and logistic regression analyses. The analysis was carried out using SPSS® version 20.

## Results

Table 1 contains respondent demographic characteristics. A total of 7080 individuals aged 18–99 years were included in data analysis. 44.9 % of respondents were males, and 55.1 % were females. Mean age was 37.27 (SD = 15.11). 38.4 % of respondents were young adults aged 18–29 years. 76.3 % were married. 40.8 % were working 15 h or more per week. 17.9 % had with a high standard of living. 8.4 % of respondents participated in community, social, and cultural events. 78.3 % of respondents attended or participated in religious activities or joined religious groups. 83.4 % of respondents regularly followed the mass media. 19.0 % were rural residents.

**Table 1:** Respondent demographic characteristics characteristics

Item	Number (*n*)	Percentage (%)
Sex		
Male	3176	44.9
Female	3904	55.1
Age groups		
18–29	2716	38.4
30–39	1772	25.0
40–49	1121	15.8
50–59	765	10.8
60 and above	706	10.0
Marital status		
Married	5399	76.3
Not married	1301	18.3
Widowed, divorced or separated	380	5.4
Labor status		
Working 15 h or more.	2891	40.8
Household work	2721	38.4
Do not work	1468	20.7
Standard of living		
Low	2284	32.3
Medium	3530	49.8
High	1266	17.9
Community participation	595	8.4
Performed religious activities	5547	78.3
Follow up mass media	5906	83.4
Type of locality		
Urban	5001	70.6
Rural	1342	19.0
Camp	737	10.4
Region		
West Bank	4686	66.2
Gaza Strip	2394	33.8

33.8 % of respondents reported low levels of well-being (ill-being) with a mean level of 58.0 (on a scale from 0 to 100, Table 2). No significant differences in well-being were observed by sex. 33.8 % of respondents aged 18–29 years reported low levels of well-being compared to 37.4 % among respondents aged 60 years and above (*p* < .05). 33.0 % of married respondents reported low levels of wellbeing compared to 41.6 % of widowed, divorced or separated (*p* < .05). 31.2 % of respondents who work 15 h or more per week reported low levels of well-being compared 37.8 % of respondents who do not work (*p* < .05). 41.6 % of respondents with low standards of living reported low levels of well-being compared to 25.2 % among respondents with high standards of living (*p* < .05). 28.4 % of respondents who participate in community, cultural, and social events reported low levels of well-being compared to 34.3 % of respondents who do not participate (*p* < .05). 33.1 % of respondents who participate in religious activities reported low levels of well-being compared to 36.5 % of those who do not participate (*p* < .05). 34.4 % of respondents who regularly follow the mass media reported low levels of well-being compared to 31.1 % of those who do not follow (*p* < .05). 30.6 % of rural residents reported low levels of well-being compared to 38.8 % of refugee camp residents (*p* < .05).

**Table 2:** Association of well-being with the independent variables

Variable name (*n*)	Low levels of well-being (%)	Moderate to high levels of well-being (%)	Chi square *P*-value
Well-being (7080)	33.8	66.2	
Age groups			
18–29 (2716)	33.8	66.2	
30–39 (1772)	35.7	64.3	.004
40–49 (1121)	31.5	68.5	
50–59 (765)	29.8	70.2	
60 and above (706)	37.4	62.6	
Marital status			
Married (5399)	33.0	67.0	
Not married (1301)	35.1	64.9	.002
Widowed, divorced or separated (380)	41.6	58.4	
Labor status			
Working 15 h or more (2891)	31.2	68.8	.000
Household worker (2721)	34.4	65.6	
Don’t work (1468)	37.8	62.2	
Standard of living			
Low (2284)	41.6	58.4	
Medium (3530)	31.9	68.1	.000
High (1266)	25.2	74.8	
Community participation			
Yes (595)	28.4	71.6	.002
No (6485)	34.3	65.7	
Attend religious activities			
Yes (5547)	33.1	66.9	.006
No (1533)	36.5	63.5	
Follow up mass media			
Yes (5906)	34.4	65.6	.016
No (1174)	31.1	68.9	
Type of locality			
Urban (5001)	34.0	66.0	
Rural (1342)	30.6	69.4	.001
Camp (737)	38.8	61.2	
Region			
West Bank (4686)	32.4	67.6	.000
Gaza Strip (2394)	36.7	63.3	

Table 3 shows the results of multivariate binary logistic regression with Well-being used as the dependent variable. The results indicate that those widowed, divorced, or separated were more likely to report low levels of well-being compared to those married [OR = 0.76, 95 % CI (0.60–0.96)]. Respondents who reported that they did not work were more likely to report low levels of well-being compared to respondents working 15 h or more per week [OR = 0.78, 95 % CI (0.67–0.91)]. people with high standard of living were more likely to report high levels of well-being compared to people with low standards of living [OR = 2.10, 95 % CI (1.80–2.46)]. Respondents who reported that they participate in community, cultural, and social events were more likely to report high levels of well-being compared to those who do not participate [OR = 1.27, 95 % CI (1.05–1.54)]. Respondents who reported not regularly following up the mass media were more likely to report high levels of well-being compared to those who regularly followed [OR = 1.18, 95 % CI (1.03–1.36)]. Residents of rural areas were more likely to report high levels of well-being compared to people living in Palestinian refugee camps [OR = 1.35, 95 % CI (1.11–1.60)].

**Table 3:** Multivariate binary logistic regression for well-being among Palestinian population

Variable^a^		*P* value	Adjusted OR	95 % C.I. for OR	
				Lower	Upper
Marital status	Married	1			
	Not married	0.18	0.90	0.78	1.05
	Widowed, divorced or separated	0.02	0.76	0.60	0.96
Labor status/Working	Working 15 h or more	1			
	Household worker	0.11	0.87	0.73	1.03
	Do not work	0.00	0.78	0.67	0.91
Standard of living	Low	1			
	Medium	0.00	1.51	1.35	1.69
	High	0.00	2.10	1.80	2.46
Community participation		0.01	1.27	1.05	1.54
Religious activities		0.00	1.25	1.10	1.42
Mass media		0.02	1.18	1.03	1.36
Type of locality	Camp	1			
	Urban	0.08	1.16	0.98	1.36
	Rural	0.00	1.35	1.11	1.60

^a^Variables entered into the model include: Age, sex, marital status, Labor status, standard of living, community participation, religious activities, mass media, and type of locality

## Discussion

In this study, we measured the prevalence of well-being using a representative sample of the adult Palestinian population, and investigated the effects of selected demographic and socio-economic factors on wellbeing. Overall, 33.8 % of the respondents reported low levels of well-being (ill-being).

The study found no effects of age on well-being. This finding is supported by findings from other studies [19]. Indeed, a meta-analysis of 119 studies conducted before 1980 for age and well-being found that the correlation between age and well-being is close to zero, and that age cannot explain more than 1 % of the total variance [20]. The stability of life satisfaction could be explained by the ability of people to adapt themselves to the process of aging and to change their goals and aspirations to suit their situation across their life span [4].

This study showed no differences in subjective well-being between the sexes. This finding is supported by the literature with several large scale surveys finding little evidence of gender differences in subjective well-being [21]. In fact, a meta-analysis of 300 empirical surveys conducted in various parts of the world provided little evidence of gender differences in subjective well-being [22]. Although some studies showed higher levels of well-being among men, and some others among women [6], the differences detected were always small [23].

The widowed, divorced, or separated respondents were found to be less likely to report high levels of well-being compared to married respondents. Interestingly, marriage -as reported by many large scale studies- is one of the most important predictors of subjective well-being even when other variables such as education and income were controlled for [24]. The positive impact of marriage on subjective well-being could be explained by the fact that marriage provides people with some sort of social support, neutralizes daily stressors, gives people a sense of belonging and purpose, and can leads to social integration [25].

Respondents who reported not working were less likely to report high levels of well-being compared to respondents working 15 h or more per week. This finding is supported by findings of other studies with the unemployed found to be the least happy sector of the society even when other variables were controlled for [23]. Indeed, some researchers found that unemployment does not allow people to achieve prosperity and develop their potentials [26]. Furthermore, working a job is noted as resulting in a better social contact, gives people resources, respect and value within the society [4].

Respondent Standard of Living were found to be strongly associated with well-being. Respondents with high Standards of Living were 2.1 times as likely to report high levels of well-being compared to people with low Standards of Living. This finding is consistent with other studies which revealed better levels of well-being among people with higher socioeconomic status [27]. Recently, Standard of Living has been increasingly used instead of income to assess levels of well-being [28]. Higher Standard of Living is understood to mean higher financial satisfaction, better quality of health services and goods, better education, housing, safety, material prosperity, greater optimism, and social and political freedom [29, 30], all of which assist in buffering stress, leading to comfort, prevalence of positive emotions, and physical as well as mental satisfaction.

Respondents who reported participating in community, cultural, and social events were 1.3 times as likely to report high levels of well-being compared to those who reported not participating. This finding is consistent with the literature that showed some positive impact of participation in social activities on subjective well-being [23]. In fact, research in this field indicates that social contact increases happiness and well-being, and changes in social contact can cause a subsequent change in well-being, even when socioeconomic and other health related variables were controlled for [30]. The reason behind this could be attributed to role of community participation in empowering people, and break their isolation [31]. Furthermore, social interactions give people a positive sense of belonging, meaning, and social identity [32].

People who reported attending or participating in religious activities were 1.3 times as likely to report high levels of well-being compared to those who reported not participating in such activities. This finding emphasizes findings of other studies which indicate that religious people tend to have better levels of well-being compared to non-religious individuals [27]. In 2003, Clark and Lelkes reported that the positive influence of religion on subjective well-being could be attributed to capacity of religion as a potential resource to neutralize daily stressors like low income, sad events, or unemployment [1]. Also, it is believed that religious practices give some sort of social support, feeling of inclusion, increases social contact, and promotes peoples’ healthy life style [33].

Respondents who do not regularly follow the mass media were 1.2 times as likely to have high levels of well-being compared to those who regularly follow. The mass media has a great influence on peoples’ culture and norms [34]. Indeed, the media was found to affect people’s values, behaviors, beliefs, and alters their perception on the economy, culture and policy [35]. It was reported to cause some drawbacks on society such as promoting violence, aggressive behaviors, harmful advertisement such as advertising for tobacco and alcohol consumption that negatively affect peoples’ choice, behavior, physical as well as mental health, leading eventually to decrease levels of population well-being [36].

Residents of rural areas were 1.4 as likely to have high levels of well-being compared to people living in refugee camps. This finding is supported by findings of other research [37]. The literature indicated that living in camp localities is associated with higher levels of psychological distress among refugees, which could be attributed to threats to personal and family safety, restricted economic opportunities, poor housing conditions, lack of food security, great poverty [38, 39], and overcrowded places [40].

The mean level of subjective well-being found in this study was 58.0, lower than the mean score reported for Denmark (after converting the scale from 25 to 100 for comparison purposes) where the mean score in a population study was 70.0; and comparable to what was reported for Lithuania at 58.2, and slightly better than Latvia at 56.2 [41]. Overall, this study reveals low levels of well-being among the Palestinian population requiring future attention.

## Conclusions

A considerable proportion of the Palestinian population reported low levels of well-being, a finding which supports our hypothesis. Interestingly, the universal factors which were found to influence well-being in several parts of the world were also found to be of influence in the Palestinian context. The study demonstrates that employment, marriage, high standards of living, community participation, and participation in religious activities are protective against ill-being. On the other hand, following the mass media on a regular basis, and living in camp localities are likely to be risk factors for ill-being.

Policy makers need to pay more attention to the concept, its’ measurement, and its’ application in population research, and struggle to improve life circumstances which positively enhance well-being. Those include, inter alia, quality education which enables individuals to find employment, improvement of income accompanied by good quality services, material prosperity, good housing, quality health services, community participation and feeling of inclusion, good governance, freedom of choice, and democracy.

### Study limitations

This is a cross sectional study, and cannot establish causation; only associations with well-being can be ascertained as a result. In addition, contextual factors like political instability, and siege conditions which affect both physical as well as mental health of the population were not investigated.

## Appendix

**1- Well-being scale questions**

1. Are you happy and in good mood during the past two weeks?
2. Do you feel calm and relaxed during the past two weeks?
3. Do you feel energetic during the past two weeks?
4. Do you wake up active and comfortable during the past two weeks?
5. Are your days were filled with things of interest to you in the past two weeks?

**2- Standard of living scale questions:**

1. Do you have goods or services to the following family/Private car?
2. Do you have goods or services to the following family/Satellite TV
3. Do you have goods or services to the following family/Solar heating of water?
4. Do you have goods or services to the following family Vacuum cleaner?
5. Do you have goods or services to the following family/Home Library?
6. Do you have goods or services to the following family/PC?
7. Do you have goods or services to the following family/Palestinian. Internet services line
8. Do you have goods or services to the following family/Microwave?
9. Do you have goods or services to the following family/DVD player?
10. Do you have goods or services to the following family/Air Conditioning?

## References

[1] Diener E, Seligman MEP (2004). Beyond money: Toward an economy of well-being. Psychol Sci Public Interest.

[2] World Health Organization (1948). WHO Constitution.

[3] Hervás G, Vázquez C (2013). Construction and validation of a measure of integrative well-being in seven languages: The Pemberton Happiness Index. Health Qual Life Out.

[4] Diener E, Suh EM, Lucas RE, Smith HL (1999). Subjective well-being: Three decades of progress. Psychol Bull.

[5] Taggart F, Friede T, Weich S, Clarke A, Johnson M, Stewart-Brown S (2013). Cross cultural evaluation of the Warwick-Edinburgh mental well-being scale (WEMWBS) -a mixed methods study. Health Qual Life Out.

[6] Huppert FA (2009). Psychological well-being: Evidence regarding its causes and consequences. Appl Psychol Health Well Being.

[7] La Placa V, Knight A (2014). Well-being: Its influence and local impact on public health. Public Health.

[8] Ryff CD, Keyes CLM (1995). The structure of psychological well-being revisited. J Pers Soc Psychol.

[9] Diener E, Lucas RE, Oishi S, Snyder CR, Lopez SJ (2002). Subjective well-being: The science of happiness and life satisfaction. Handbook of positive psychology.

[10] Kahneman D, Deaton A (2010). High income improves evaluation of life but not emotional well-being. Proc Natl Acad Sci U S A.

[11] Falah G (2003). Dynamics and patterns of the shrinking of Arab lands in Palestine. Polit Geogr.

[12] Batniji R, Rabaia Y, Nguyen-Gillham V, Giacaman R, Sarraj E, Punamaki RL (2009). Health as human security in the occupied Palestinian territory. Lancet.

[13] Kondylis F (2010). Conflict displacement and labor market outcomes in post-war Bosnia and Herzegovina. J Dev Econ.

[14] Palestinian Central Bureau of Statistics (2014). Time use survey, 2012/2013 User’s Guide.

[15] Sibai AM, Chaaya M, Tohme RA, Mahfoud Z, Alamin H (2009). Validation of the Arabic version of the 5-item WHO Well being index in elderly population. Int J Geriatr Psychiatry.

[16] Topp CW, Østergaard SD, Søndergaard S, Bech P (2015). The WHO-5 well-being index: A systematic review of the literature. Psychother Psychosom.

[17] World Health Organisation (1998). Well-being measures in primary health care/The Depcare project.

[18] Palestinian Central Bureau of Statistics (2008). The Household Expenditure and Consumption Survey (PECS) -2007.

[19] Diener E (1984). Subjective well-being. Psychol Bull.

[20] Stock WA, Okun MA, Haring MJ, Witter RA, Light RJ (1983). Age and subjective well-being: A meta-analysis. Evaluation studies: Review annual.

[21] Helliwell JF (2003). How’s life? Combining individual and national variables to explain subjective well-being. Econ Model.

[22] Pinquart M, Sorensen S (2001). Gender differences in self-concept and psychological well-being in old age: A meta-analysis. J Gerontol B Psychol Sci Soc Sci.

[23] Campbell A, Converse PE, Rodgers WL (1976). The quality of American life: Perceptions, evaluations, and satisfactions.

[24] Glenn ND, Weaver CN (1979). A note on family situation and global happiness. Soc Forces.

[25] Shapiro A, Keyes CLM (2007). Marital status and social well-being: Are the married always better off?. Soc Indic Res.

[26] Huppert FA, Whittington JE (2003). Evidence for the independence of positive and negative well-being: Implications for quality of life assessment. Br J Health Psychol.

[27] Dolan P, Peasgood T, White M (2008). Do we really know what makes us happy? A review of the economic literature on the factors associated with subjective well-being. J Econ Psychol.

[28] Kahneman D, Krueger AB (2006). Developments in the measurement of subjective well-being. J Econ Perspect.

[29] Baikova YI, Vardiashvili NN (2015). To the problem of rise in the standard of living of population. Asian Soc Sci.

[30] Bradburn NM (1969). The structure of psychological well-being.

[31] Christens BD (2012). Targeting empowerment in community development: A community psychology approach to enhancing local power and well-being. Community Dev J.

[32] Haslam SA, Jetten J, Postmes T, Haslam C (2009). Social identity, health and well-being: An emerging agenda for applied psychology. Appl Psychol Int Rev.

[33] Ellison CG (1991). Religious involvement and subjective well-being. J Health Soc Behav.

[34] Akbar MW. Cultural invasion of western media and Muslim societies. Global Media Journal: Pakistan edition. 2009;2(2). http://www.aiou.edu.pk/gmj/CULTURAL%20INVASION%20OF%20WESTERN%20MEDIA%20AND%20MUSLIM%20SOCIETIES-4.asp. Accessed 25 Mar 2016.

[35] Khan AY, Razi A, Mirza R, Mazhar S, Amjad A, Shafique U (2013). Impact of mass media in Pakistan on social, ethical, and economic grounds. Int J Eco Res.

[36] Mehraj HK, Bhat AN, Mehraj HR (2014). Impacts of media on society: A sociological perspective. Inter J Humanit Soc Sci Invent.

[37] Habib RR, Basma SH, Yeretzian JS (2006). Harboring illnesses: On the association between disease and living conditions in a Palestinian refugee camp in Lebanon. Int J Environ Health Res.

[38] Alnsour J, Meaton J (2014). Housing conditions in Palestinian refugee camps, Jordan. Cities.

[39] Khawaja M (2003). Migration and the reproduction of poverty: The refugee camps in Jordan. Int Migr.

[40] Giacaman R, Mataria A, Nguyen-Gilham V, Safieh RA, Stefanini A, Chatterji S (2007). Quality of life in the Palestinian context: An inquiry in war-like conditions. Health Policy.

[41] European Foundation for the Improvement of Living and Working Conditions. Quality of life in Europe: Subjective well-being. Dublin: Author; 2013.

